# CRISPR/Cas9 Genome Engineering in Non‐Conventional Oleaginous Yeasts: Applications, Challenges, and Prospects

**DOI:** 10.1002/yea.70015

**Published:** 2026-03-19

**Authors:** Rodrigo Gonçalves Dias, Fernanda Pinheiro Moreira Freitas, Eduardo Luís Menezes de Almeida, Luciano Gomes Fietto, Agustin Zsögön, Wendel Batista da Silveira

**Affiliations:** ^1^ Department of Microbiology Universidade Federal de Viçosa Viçosa Minas Gerais Brazil; ^2^ Department of Biochemistry and Molecular Biology Universidade Federal de Viçosa Viçosa Minas Gerais Brazil; ^3^ Department of Plant Biology National Institute of Science and Technology on Plant Physiology Under Stress Conditions, Universidade Federal de Viçosa Viçosa Minas Gerais Brazil

**Keywords:** biorefineries, Candida spp, *Cutaneotrichosporon oleaginosus*, fatty acids, metabolic engineering, *Rhodotorula toruloides*

## Abstract

Given the biotechnological potential of yeast‐derived oils for oleochemical production, genes encoding lipid metabolism enzymes are key targets for metabolic engineering. Genetic engineering tools such as Clustered Regularly Interspaced Short Palindromic Repeats (CRISPR)/Cas9, Transcription Activator‐Like Effector Nucleases (TALENs), Zinc‐Finger Nucleases (ZFNs), RNA interference (RNAi), and integrative plasmids can be used to modulate fatty acid biosynthesis and optimize lipid production. Among them, the CRISPR/Cas9 system, recognized for its simplicity and efficiency, has been deployed as a tool to create oleaginous yeast strains with high lipid productivity and features suitable for application in biorefineries. Species such as *Cutaneotrichosporon oleaginosus*, *Rhodotorula toruloides*, *Candida* spp., and *Yarrowia lipolytica* have already been engineered using CRISPR/Cas9 to enhance the production of fatty acids and their derivatives. However, designing and constructing an efficient CRISPR/Cas9 platform for oleaginous yeasts faces several hurdles, including low transformation efficiency, difficulties in expressing Cas9 and sgRNAs efficiently and consistently, the lack of well‐characterized promoters, limited availability of PAM sequences, and poorly understood DNA repair mechanisms. Here, we address the application of the CRISPR/Cas9 system in oleaginous yeasts, laying out the challenges to developing efficient platforms and highlighting key trends in the field. We compare and discuss alternative CRISPR‐Cas9 expression strategies to provide an overview of the current landscape and support the development of new approaches.

## Summary

- A chronological review of the main milestones and advances in the use of the tool in different oleaginous yeast species is presented.
- The systems already developed and optimized for oleaginous yeasts are described, with emphasis on their applications in metabolic engineering.
- Technical limitations, knowledge gaps, and future opportunities to expand the tool's applicability in oleaginous yeasts are discussed.

## Graphical abstract

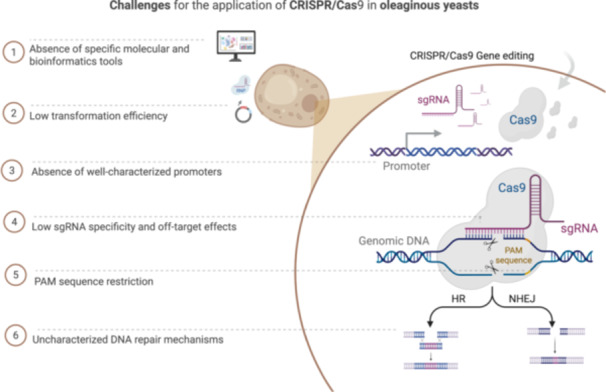

## 1 Introduction

Lipids play key metabolic, signaling, and structural roles in microbial cells (Fahy et al. 2009). As essential cellular components, these molecules are present in all microorganisms, comprising around 6%–10% of their dry weight. Some microorganisms, including both eukaryotes and prokaryotes, can produce and accumulate over 20% of their dry weight in lipids, and are thus classified as oleaginous (Thorpe and Ratledge 1972; Ratledge and Wynn 2002; Abeln and Chuck 2021). Microbial lipids have wide‐ranging industrial applications, particularly as ingredients in the food industry, as substrates for the production of fatty acid‐derived biofuels and oleochemicals, drug carriers, active compounds, and supplements in pharmaceutical and cosmetic industries (Bharathiraja et al. 2017). Among oleaginous microorganisms, yeasts stand out for industrial lipid production due to their favorable characteristics, such as high cell growth rates, high lipid productivity, light‐independent growth, and the ability to metabolize diverse carbon sources, including sugars derived from lignocellulosic biomasses (Abeln and Chuck 2021). This latter capability is pivotal to boost the development of sustainable biorefineries by valorizing industrial and agricultural byproducts, promoting the circular bioeconomy (Sawangkeaw and Ngamprasertsith 2013; Abeln and Chuck 2021).

The lipids produced by oleaginous yeasts predominantly consist of triacylglycerols, of which C:16 and C:18 fatty acids are the most abundant, generally in unsaturated forms (Athenstaedt et al. 2006). Lipid biosynthesis in yeasts occurs through de novo and ex novo pathways (Ratledge and Wynn 2002). In the de novo pathway, prevalent in non‐oleaginous yeasts, the acetyl‐CoA precursor is primarily derived from glycolysis and pyruvate metabolism. In the most studied oleaginous yeasts, acetyl‐CoA can also be formed from citrate transported from the mitochondria to the cytosol, where it is cleaved by ATP‐citrate lyase, an enzyme absent in non‐oleaginous yeasts (Botham and Ratledge 1979; Abeln and Chuck 2021). The ex novo pathway involves the uptake of exogenous fatty acids by the cell, which are then used for lipid synthesis or cellular growth. Unlike the de novo pathway, lipid accumulation and cell growth occur simultaneously in the ex novo pathway (Ratledge and Wynn 2002).

Due to the industrial potential of yeast oil, enzymes involved in lipid metabolism have become prime targets for metabolic engineering (Abeln and Chuck 2021). Genetic manipulation tools such as Clustered Regularly Interspaced Short Palindromic Repeats (CRISPR)/Cas9, Transcription Activator‐Like Effector Nucleases (TALENs), Zinc‐Finger Nucleases (ZFNs), RNA interference (RNAi), and integrative plasmids can target genes encoding specific enzymes in the fatty acid biosynthesis pathway to modulate the oleaginous phenotype, enhance lipid production, and tailor fatty acids for various industrial applications (Shan et al. 2021). The CRISPR/Cas9 system stands out among genetic manipulation tools due to its simplicity and efficiency. CRISPR employs an easily designed single‐guide RNA (sgRNA) to direct the Cas9 endonuclease to a target gene, enabling precise and multiplexed edits (Shan et al. 2021; Chaudhuri et al. 2022) (Table 1).

**Table 1:** Application of CRISPR in oleaginous yeasts.

Species	CRISPR system	Delivery of components	Genetic modification	Repair mechanism	Objective/Product	References
*Cutaneotrichosporon oleaginosus*	CRISPR/Cas9	Direct transformation with ribonucleoprotein complex (RNP); Transformation of sgRNA and Cas9 mRNA;	Deletion of the *URA5* gene and modification of the Δ‐9 and Δ‐12 desaturase genes.	HDR	Protocol standardization and modification of fatty acid profile	Shaigani et al. (2023)
*Rhodotorula toruloides* IFO0880	CRISPR/Cas9	Integrative plasmid containing the sgRNA and Cas9 gene	Silencing of *URA3* and *CAR2* genes	NHEJ	Protocol standardization	Otoupal et al. (2019)
*Rhodotorula toruloides* NP11	CRISPR/Cas9	Cas9 transformation by *Agrobacterium tumefaciens* (ATMT); transformation with Cas9 for sgRNA expression and DNA template	Silencing of *CRTI* and *CAR2* genes	NHEJ; HDR	Protocol standardization	Jiao et al. (2019)
*Rhodotorula toruloides* NP11	CRISPR/Cas9	Cas9 transformation by ATMT; transformation with Cas9 for sgRNA expression and DNA template	Silencing of *CRTI* and *CRTYB* genes	NHEJ	Protocol standardization	Schultz et al. (2019)
*Rhodotorula toruloides* NP11	CRISPR/Cas9	Cas9 transformation by *Agrobacterium tumefaciens* (ATMT); transformation with Cas9 for sgRNA expression	Silencing of *Ldp1* and *Cals* genes	NHEJ	Reduction of lipid production and evaluation of metabolic flux for carotenoid synthesis	Jiao et al. (2021)
*Rhodotorula toruloides* IFO0880	CRISPR/Cas9	Cas9 transformation by ATMT; transformation with Cas9 for sgRNA expression and DNA template	*G6PD, ACL1, ACC1, LRO1, DGA1, TGL2, FAA1, ARE1, FAR, PXA1, FAO*	NHEJ; HR	Increasing fatty acid production through the manipulation of lipid metabolism genes	Schultz et al. (2022)
*Rhodotorula* spp. U13N3	CRISPR/Cas9	Cas9 transformation by ATMT; transformation with Cas9 for sgRNA expression	Silencing of *ATG15* and *ACOX2* genes	NHEJ	Decreased triacylglycerol catabolism to increase lipid production	Song et al. (2023)
*Rhodotorula toruloides* IFO0880	CRISPR/Cas9	Integrative plasmid containing the sgRNA and Cas9 gene	Silencing of *CIT2*, *MLS1*, *PYC1*, *DGA1*, and *LRO1* genes	NHEJ	Production of triacetic acid lactone	Cao et al. (2022)
*Rhodotorula toruloides* IFO0880	CRISPR/Cas9	Integrative plasmid containing the sgRNA and Cas9 gene	Silencing of *RTO4_8975* genes	NHEJ	Production of 3‐hydroxypropionic acid	Liu, Hwang, et al. (2024)
*Rhodotorula toruloides* NP11	CRISPR/Cas9	Cas9 transformation by ATMT; transformation with Cas9 for sgRNA expression	Silencing of *CRT* and *LDP1*genes	NHEJ	α‐terpineol production	Lyu et al. (2024)
*Rhodotorula toruloides 2.1389 (RT1389)*	CRISPR/Cas9‐assisted Cre recombination (CACR)	Cas9 and CRE transformation by ATMT; transformation with Cas9 for sgRNA expression	Iterative editing in ergothioneine biosynthesis and precursor genes	NHEJ	Ergothioneine production	Liu, Xiang, et al. (2024)
*Rhodotorula kratochvilovae YM25235*	CRISPR/Cas9	Cas9 transformation by ATMT; transformation with Cas9 for sgRNA expression	*RKCrtYB* gene silencing	NHEJ	Study of carotenoid metabolism	Guo et al. (2022)
*Rhodotorula toruloides* NP11	CRISPR/Cas9	Transformation by *Agrobacterium tumefaciens* (ATMT);	*Silencing of the genes HFD1, FAA1–FAA6, PEX10, AOX1, PAH1, DGA1, LRO1 and ALK1/ALK2*	NHEJ	Increase lipid production	Yu et al. (2025)
*Rhodotorula toruloides* IFO0880	CRISPR/Cas9	Transformation of plasmids and Cas9 by the lithium chloride method	*Silencing of PK, ACL, or cMAE genes*	NHEJ	Influence of silencing the pyruvate kinase, ATP‐citrate lyase, and cytosolic malic enzyme genes on the oleaginous phenotype	Reķēna et al. (2025)
*Candida* spp. species	CRISPR/Cas9	—	—	—	Review (2015−2021)	Uthayakumar et al. (2021)
*Candida tropicalis* Cu‐206	CRISPR/dCas9	Transformation of plasmids and Cas9 by the lithium chloride method	*GFP3*, *URA3, ADE2 and ERG9 Candida tropicalis* Cu‐206	—	Standardization of a CRISPRi system and regulation of β‐carotene biosynthesis	Li et al. (2022)
*Candida tropicalis* Cu‐206	CRISPR/Cas9	Transformation of plasmids and Cas9 by the lithium chloride method	Integration of *ZSS1, NADH‐HMGR, ERG10, ERG13, ERG13, ERG12, ERG8, ERG19, IDI1*, and *ERG20* genes	NH	Heterologous production of α‐humulene	Zhang et al. (2022)
*Candida tropicalis* Cu‐208	CRISPR/Cas9	Transformation of plasmids and Cas9 by the lithium chloride method	Integration of *CBTS1*, *ERG20*, *BTS1* and *CBTS1*	NHEJ	Heterologous production of cembratrien‐ol	Zhang et al. (2024)
*Candida tropicalis DC03*	CRISPR/Cas9 and CRISPR/dCas9	Transformation of plasmids and Cas9 by the lithium chloride method	Integration and modulation of the genes *CtERG9*, *CtERG9*, *ScERG9*, *ERG20*, *DPP1*, *BTS1*, and *ERG1*	NHEJ	Heterologous production of esqualeno	Wei et al. (2024)
*Candida viswanathii* ATCC 20962	CRISPR/Cas9	Transformation by electroporation of plasmids and integration of Cas9	Integration of *CYP52A19*, *CPRb*, *FAO2*, and *POS5* genes	NHEJ	Increased production of dodecanedioic acid	Pham et al. (2023)

CRISPR/Cas9‐mediated gene editing was successfully applied for the first time in yeasts using the model *Saccharomyces cerevisiae* in 2013 (DiCarlo et al. 2013), and was henceforth widely adopted in this species (Stovicek et al. 2017). This milestone spurred the adaptation of the technique for other yeasts of interest, such as *Candida albicans* (Vyas et al. 2015), *Yarrowia lipolytica* (Schwartz et al. 2016), and *Komagataella phaffii* (Weninger et al. 2016). Since then, the CRISPR/Cas9 system has been established as an efficient tool for the genetic engineering of yeasts with high biotechnological potential, including oleaginous species. The CRISPR/Cas9 platform can be used to edit key genes in fatty acid biosynthesis, rewiring metabolic fluxes, and customize lipid compounds. It also facilitates the introduction of heterologous metabolic pathways, optimization of alternative substrates such as sugar constituents of lignocellulosic biomasses, reduction of undesirable fermentation byproducts, and functional investigation of lipid metabolism‐related genes (Table 1). These advancements have contributed to expanding the biotechnological potential of oleaginous yeasts for various industrial applications.

This review focuses on exploring the application of the CRISPR/Cas9 system in oleaginous yeasts, addressing the challenges for the development of efficient platforms, as well as highlighting some future prospects in the field. We also address the delivery strategies of the system components, discussing their relevance for specific applications. Next, we detail the application of CRISPR/Cas9 in oleaginous yeasts, focusing on the genera *Cutaneotrichosporon*, *Rhodotorula,* and *Candida*. Moreover, we analyze the challenges associated with the use of this technology in these yeasts, including limitations in transformation efficiency, promoter selection, DNA repair mechanisms, and available molecular tools. Lastly, future perspectives to overcome these barriers are discussed.

## 2 CRISPR/Cas9 Components and Their Delivery to Target Cells

**Figure 1:**
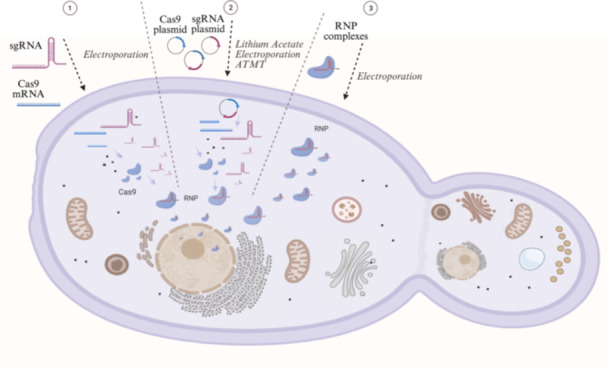
Schematic representation of CRISPR/Cas9 delivery methods for genome editing in yeast cells, illustrating three primary approaches: (1) co‐delivery of sgRNA and Cas9 mRNA for transient protein expression, (2) plasmid‐based delivery of both components for stable or transient expression, and (3) direct introduction of pre‐formed ribonucleoprotein (RNP) complexes (sgRNA+Cas9) for immediate editing activity. The more common yeast transformation methods include electroporation, lithium acetate treatment, and *Agrobacterium tumefaciens*‐mediated transformation (ATMT).

For precise genome editing, the CRISPR system relies on two key components: the sgRNA and a Cas9 endonuclease. The sgRNA contains a ~20‐nucleotide sequence that directs the complex to the specific DNA target, while the Cas9 protein mediates site‐specific double‐strand breaks, activated by a protospacer adjacent motif (PAM) in the target DNA (Gasiunas et al. 2012; Jinek et al. 2012). Several strategies are employed to deliver CRISPR/Cas9 components into yeast cells (Figure 1) (Shaigani et al. 2023). The large size of Cas9 (158.9 kDa for spCas9; encoded by a ~4 kb gene) poses challenges for transformation. Delivery methods include integrative or episomal plasmids or direct introduction of Cas9 mRNA (Shaigani et al. 2023; Wilbie et al. 2019). The sgRNA can be introduced as synthetic single‐stranded oligonucleotides or expressed from plasmids. Both elements may also be co‐delivered on a single plasmid or as preassembled ribonucleoprotein (RNP) complexes, which require only a nuclear localization signal (NLS) to function. In oleaginous yeasts, the most frequently used transformation methods include electroporation, lithium acetate‐based protocols, and *Agrobacterium tumefaciens*‐mediated transformation (ATMT) (Figure 1).

The NLS is a short amino acid sequence (frequently rich in K and R) that enables active transport of proteins like Cas9 into the nucleus via importins. Two main classes of NLS exist: monopartite (a single basic stretch) and bipartite (two clusters of basic residues separated by a spacer). The nuclear import process comprises three steps: (i) binding of the NLS‐bearing Cas9 protein to importin in the cytoplasm; (ii) translocation through nuclear pores; and (iii) dissociation of the complex within the nucleus, releasing Cas9 to execute genome editing (Lu et al. 2021). Following DNA cleavage, two major repair mechanisms compete: non‐homologous end joining (NHEJ) and homology‐directed repair (HDR). NHEJ typically results in random insertions or deletions, which may disrupt gene function. HDR, in contrast, allows precise genome modifications using an exogenous DNA template, enabling site‐specific insertions or replacements (Jiang and Doudna 2017; Raschmanová et al. 2018; Yang and Blenner 2020). The balance between these pathways is influenced by factors such as the cell cycle phase, DNA repair gene repertoire, and experimental conditions. NHEJ predominates during G1, when homologous templates are absent, whereas HDR is favored in S/G2, when sister chromatids are available. Genetic manipulations can modulate pathway preference: deletion of KU70/KU80 enhances HDR efficiency, while loss of RAD52 homologs impairs HDR, shifting repair toward alternative mechanisms (Åström et al. 1999; Aylon and Kupiec 2004).

## 3 Application of CRISPR/Cas9 in Oleaginous Yeasts

*Cutaneotrichosporon* spp., *Rhodotorula* spp., *Lipomyces* spp., *Yarrowia* spp., and *Candida* spp. are the oleaginous yeasts most studied, aiming at application in bioprocesses (Abeln and Chuck 2021). There are CRISPR/Cas9 systems available for *Cutaneotrichosporon* spp., *Rhodotorula* spp., *Y. lipolytica,* and *Candida* spp. but not for *Lipomyces* spp. Genetic manipulation mediated by CRISPR/Cas9 systems in oleaginous yeasts has various goals, such as increasing lipid accumulation, customizing the fatty acid profile for diverse applications, enhancing the ability to tolerate environmental and industrial stresses, and improving the metabolism of complex substrates, thereby increasing their potential for use in biorefineries (Table 1). The following section discusses different CRISPR/Cas9 research studies in the main groups of oleaginous yeasts. Due to the large number of available studies on the application of CRISPR/Cas9 in *Y. lipolytica*, a review article on this species is being prepared by our research group.

### 3.1 Cutaneotrichosporon oleaginosus

*C. oleaginosus* is an oleaginous yeast that uses hexoses and pentoses and accumulates lipids up to 80% of its dry cell weight (Otoupal et al. 2019; Stellner et al. 2023). It grows efficiently on lignocellulosic hydrolysates and demonstrates greater tolerance to inhibitors such as furfural and hydroxymethylfurfural compared to other yeasts (Abeln and Chuck 2021). However, its genetic manipulation is hampered by the lack of characterized promoters and the absence of a plasmid‐based expression system (Stellner et al. 2023). Until recently, transformation relied on ATMT or electroporation with random DNA integration, both of which have limitations for targeted genome editing. A recent study established a CRISPR/Cas9‐based genome editing protocol for *C. oleaginosus* using spheroplasting and an enzymatic hydrolysis system derived from *Trichoderma reesei* (Shaigani et al. 2023). Three delivery strategies were tested using the *URA5* gene as a model target: (i) Cas9 mRNA + sgRNA + ssDNA repair template, (ii) Cas9 protein RNP complex + ssDNA, and (iii) Cas9 nickase (nCas9) RNP with dual sgRNAs. The editing efficiencies were 100%, 20%, and 75%, respectively. This approach enabled the precise knockout of *URA5* and facilitated the generation of uracil auxotrophic strains.

After standardizing the proposed CRISPR/Cas9 strategies, a methodology with three approaches was carried out for the regulation of Δ9 desaturase (*D9FAD*) and Δ12 desaturase (*D12FAD*) genes in an auxotrophic *C. oleaginosus* strain: positive regulation through overexpression, negative regulation based on promoter swapping, and complete knockout of the target genes. In the fatty acid composition analysis of the wild‐type (WT) strain, 30% palmitic acid (C16:0), 8% stearic acid (C18:0), 52% oleic acid (C18:1), 9% linoleic acid (C18:2), and 1% linolenic acid (C18:3) were observed. The D9OE strain, which underwent positive regulation of *D9FAD*, showed a significant increase in C18:1 to 55% and a reduction in C18:0 (*p* < 0.001). The D12OE strain, with positive regulation of *D12FAD*, showed a small increase in C18:2. Additionally, the regulated strains with expression levels controlled by AKRpr and TEFpr promoters for D9FAD (AKRp‐D9 and TEFp‐D9) exhibited a significant increase in saturated fatty acids (64% and 62% of TFA, respectively) and a reduction in C18:1 (25% and 29% of TFA, respectively). Replacing the native D12FADpr also had an effect, with a slight increase in C18:2, indicating that AKRpr and TEFpr are more efficient than the native promoter for *D12FAD*. The *D12FAD* knockout (ΔD12 strain) resulted in the complete absence of C18:2 and C18:3, while C18:1 increased to 64% of TFA. These results highlight that both *D9FAD* and *D12FAD* genes are suitable targets to control fatty acid composition, especially by increasing the proportion of saturated and monounsaturated fatty acids, which may be advantageous for industrial applications (Shaigani et al. 2023).

### 3.2 Rhodotorula Spp

Yeasts of the genus *Rhodotorula* spp., particularly *Rhodotorula toruloides*, have attracted considerable attention due to their remarkable capacity to accumulate high levels of lipids and carotenoids. This species is metabolically versatile, capable of assimilating a wide range of carbon sources, including those derived from lignocellulosic hydrolysates, and sustaining growth in high‐cell‐density cultures. In addition, several *R. toruloides* strains exhibit high tolerance to osmotic stress. These physiological traits, combined with their potential for the sustainable production of value‐added bioproducts, have driven increasing efforts toward the development of efficient and specific CRISPR/Cas9‐based genome editing strategies in *Rhodotorula* spp. (Abeln and Chuck 2021; Zhang et al. 2024).

CRISPR/Cas9‐mediated genome editing in *Rhodotorula* spp. was reported by Otoupal et al. (2019) in the haploid strain *R. toruloides* IFO0880. In this work, the authors established a functional CRISPR–Cas9 system through codon optimization of the *Streptococcus pyogenes* Cas9 (spCas9) gene and the incorporation of an NLS using the GenScript platform. Cas9 expression was driven by the strong GAPDHpr and terminated by the NOS terminator, both previously validated for high‐level expression in *R. toruloides*. The system architecture included an sgRNA expression cassette positioned upstream of the Cas9 coding sequence, consisting of a promoter, a hepatitis delta virus (HDV) ribozyme, a 20‐nucleotide guide sequence, a 76‐nucleotide Cas9‐binding scaffold, and a transcriptional terminator. For sgRNA expression, the *S. cerevisiae* RNA polymerase III SNR52pr and terminator were employed, while a nourseothricin resistance gene (NATʳ) enabled selection of transformants. Genetic constructs were assembled in *Escherichia coli* XL1‐Blue or DH5α and introduced into *R. toruloides* via lithium acetate (LiAc)‐mediated transformation after restriction enzyme digestion of plasmid constructs (HindIII for p90–p99 and p184–p190; NdeI for pGI103–pGI132). System validation was achieved through deletion of the *URA3* gene, relying predominantly on NHEJ repair. Importantly, this study also marked the beginning of systematic optimization of CRISPR performance in *R. toruloides*. Redesign of sgRNAs using sgRNA Scorer 2.0 increased editing efficiency by 14‐fold, while removal of the HDV ribozyme from the 5′ end improved efficiency by up to 26‐fold. Furthermore, replacing the heterologous SNR52pr with native *R. toruloides* tRNA promoters (tRNA^Phe and tRNA^Tyr) resulted in efficiency increases of up to 14‐fold, establishing endogenous tRNAs as highly effective drivers of sgRNA expression in this yeast (Otoupal et al. 2019).

Based on this platform, Otoupal et al. (2019) also introduced one of the earliest CRISPR multiplexing strategies in *R. toruloides*. A construct simultaneously targeting the *URA3* and *CAR2* genes—implicated in β‐carotene biosynthesis—was developed using an array configuration of multiple sgRNAs separated by tRNA sequences. This design reduced DNA burden and enabled simultaneous targeting of multiple loci, increasing the likelihood of complete gene disruption through the induction of double‐strand breaks at two sites per gene, separated by approximately 500 bp.

In parallel, Jiao et al. (2019) established an alternative CRISPR/Cas9 platform in the *R. toruloides* NP11 strain, introducing several methodological innovations. Instead of spCas9, this system employed a codon‐optimized Cas9 derived from *Staphylococcus aureus*. A key advance was the identification and use of endogenous U6pr from the NP11 strain to drive sgRNA transcription, with the U6bpr showing the highest efficiency. Cas9 expression, enhanced by NLS incorporation, was controlled by the GPDpr and introduced into NP11 via ATMT, followed by insertion of sgRNA expression cassettes. Targeting of the *CRTI* and *CAR2* genes resulted in an albino phenotype, enabling rapid phenotypic screening. This study also characterized DNA repair pathways in NP11, demonstrating NHEJ predominance, while homologous recombination (HR) occurred at low frequency (< 2%). Nevertheless, the results indicated that HDR efficiency could be improved through optimization of homology arm length and transformation parameters.

Further methodological improvements were achieved by Schultz et al. (2019), who developed a CRISPR/Cas9 system for efficient multi‐gene silencing in *R. toruloides* NP11. Extensive promoter screening revealed that the pPGK1pr yielded the highest Cas9 expression and transformation efficiency. The authors constructed a pPGK1‐Cas9‐NLS3 (Addgene plasmid #128177) cassette and integrated it into the NP11 genome via ATMT, generating a host strain with constitutive Cas9 expression. For sgRNA expression, a *S. cerevisiae* 5S rRNA–tRNA fusion promoter was adapted, and alternative ribozyme‐based architectures (HH–gRNA–HDV) were tested to enable RNAP II‐driven sgRNA expression. Knockout efficiencies exceeded 99% for reporter genes (*CRTYB*, *CRTI*), particularly when sgRNAs were expressed under the 5S rRNA promoter. To enable multiplex editing, sequential 5S‐tRNA Cas9 cassettes were integrated into the genome; insertion of a 150 bp spacer between sgRNA cassettes significantly increased double‐knockout efficiency to 78%, underscoring the importance of cassette architecture for multiplex CRISPR performance.

With the establishment of these robust platforms, subsequent studies shifted focus from methodological development to applications in metabolic engineering. Jiao et al. (2021) reported the first CRISPR/Cas9‐mediated modulation of lipid accumulation in *R. toruloides* through deletion of the lipid droplet‐associated proteins LDP1 and CALS in an NP11‐Cas9 strain. Using plasmids constructed via restriction‐free cloning and delivered by ATMT, the authors demonstrated that disruption of these structural proteins reduced lipid content by more than 40%, while simultaneously increasing carotenoid accumulation to approximately 3.0 mg/g, indicating a redirection of carbon flux from lipid storage to the terpenoid pathway. Song et al. (2023) further explored CRISPR/Cas9 to enhance lipid accumulation by attenuating lipid catabolism in *Rhodotorula* spp. U13N3. Deletion of the *ATG15* (lipase) and *ACOX2* (peroxisomal acyl‐CoA oxidase) genes, individually and in combination, resulted in significant increases in lipid production, with the double mutant showing a 67.03% increase without deleterious effects on growth. These modifications proved effective under fed‐batch and bioreactor conditions, demonstrating industrial relevance.

In *R. toruloides* IFO0880, Schultz et al. (2022) applied CRISPR/Cas9 for systematic investigation of lipid metabolism by promoting overexpression or individual deletion of 16 genes involved in precursor supply, NADPH generation, triacylglycerol synthesis, and lipid degradation. Combinatorial engineering revealed synergistic effects, particularly when overexpression of *ACC1* and *ACL1* was combined with deletion of *DGA1* and *LRO1*, substantially increasing fatty acid titers. A notable methodological advance was the introduction of CRISPR‐assisted Cre–loxP recombination (CACR) by Liu, Xiang, et al. (2024) in *R. toruloides* RT1389, enabling iterative and marker‐free genome editing. By combining CRISPR/Cas9‐mediated targeting with inducible excision of selectable markers via Cre recombinase, the authors generated scarless strains with enhanced genetic stability, culminating in high‐level ergothioneine production.

Beyond lipid engineering, CRISPR/Cas9 methodologies have been extended to the production of diverse industrial metabolites, including triacetic acid lactone (Cao et al. 2022), 3‐hydroxypropionic acid (Liu, Hwang, et al. 2024), α‐terpineol (Lyu et al. 2024), and ergothioneine (Liu, Xiang, et al. 2024). These studies increasingly employed refined CRISPR architectures, stable chromosomal integration, and modular metabolic pathway engineering.

More recently, CRISPR/Cas9 has been widely applied to the study of central carbon metabolism in *Rhodotorula toruloides*. Reķēna et al. (2025) employed a chromosomally integrated spCas9 system to generate knockouts of the *PK*, *ACL*, and *cMAE* genes in the IFO0880 strain. The results demonstrated that *ACL* is essential for both lipid biosynthesis and cellular growth, with effects strongly dependent on the carbon source, whereas *cMAE* was not critical and, under certain conditions, was even detrimental. Disruption of *PK* indicated that this enzyme is not essential for lipid synthesis, highlighting the potential of CRISPR/Cas9 as a powerful tool for detailed functional analyses of cellular metabolism.

Similarly, Yu et al. (2025) employed a previously established CRISPR/Cas9 system (Schultz et al. 2019) to perform targeted gene knockouts in *R. toruloides*, using constitutive expression of *S. pyogenes* Cas9 integrated into the NP11‐Cas9 strain. Genome editing relied predominantly on NHEJ repair, mediated by a single guide RNA per target gene, resulting in frameshift mutations and loss of function without the use of HR. CRISPR/Cas9 was applied exclusively for the disruption of endogenous genes to eliminate competing metabolic pathways, increase the availability of lipid‐derived precursors (free fatty acids, acyl‐CoA, and fatty aldehydes), and prevent consumption of the final product. Key targets included knockout of *HFD1*, which increased alkane production by up to 13‐fold via the AAR/ADO pathway, as well as deletion of multiple *FAA* genes and *PEX10*, which elevated free fatty acid pools and favored alkene production through the UndB pathway. Other genes, such as *DGA1*, *LRO1*, *ALK1*, and *ALK2*, were also evaluated to optimize metabolic flux toward hydrocarbon biosynthesis, although not all deletions resulted in positive effects. From a methodological standpoint, these studies highlight the consolidation of CRISPR/Cas9 strategies in *R. toruloides* that rely predominantly on NHEJ‐mediated knockouts, exploiting constitutive expression of *S. pyogenes* Cas9 integrated into the genome and the use of a single guide RNA per target gene. This architecture confers high robustness and operational simplicity, enabling rapid generation of loss‐of‐function mutants and facilitating functional analyses and metabolic engineering applications.

*Rhodotorula kratochvilovae* YM25235 is an oleaginous yeast capable of growing at temperatures below 15°C while producing high levels of carotenoids. To investigate the role of carotenoid biosynthesis in cold adaptation, Guo et al. (2022) established a CRISPR/Cas9 system targeting the RKCrtYB gene. A codon‐optimized *S. pyogenes* Cas9 was cloned into a *Rhodotorula*‐compatible backbone, generating the plasmid pRHCas9, which enabled stable Cas9 expression in YM25235. sgRNA expression cassettes were constructed using the endogenous U6 promoters P_RKU6a and P_RKU6b and delivered on a separate plasmid. Disruption of RKCrtYB resulted in reduced carotenoid production and impaired growth at low temperatures, highlighting the importance of carotenoid metabolism for cold tolerance in *R. kratochvilovae* (Guo et al. 2022).

### 3.3 Candida Spp

The *Candida* genus encompasses a diverse group of yeasts that exhibit both clinical relevance—such as *C. albicans*, *C. glabrata*, *C. auris*, and *C. tropicalis*—and significant biotechnological potential, exemplified by species such as *C. krusei*, *C. tropicalis*, *C. oleophila*, and *C. maltosa* (Uthayakumar et al. 2021). In this context, *C. albicans* was the first species within the genus to be genetically modified using a CRISPR/Cas9 platform (Vyas et al. 2015). Between 2015 and 2021, nine studies were published focusing on the standardization and application of the CRISPR/Cas9 system in different species of the genus, reflecting an initial phase characterized by conceptual validation of the tool and by overcoming technical challenges such as Cas9 expression, sgRNA delivery, and HR efficiency. These studies were systematically reviewed by Uthayakumar et al. (2021). Accordingly, the present review focuses on studies published after 2021, a period marked not only by efficient genome editing but also by the increasing sophistication of regulatory and metabolic engineering strategies.

A clear example of this methodological evolution is the work of Li et al. (2022), who developed for *C. tropicalis* Cu‐206 a sgRNA expression strategy based on the tRNA:gRNA platform, in addition to implementing a CRISPR interference (CRISPRi) system. The authors used an endogenous tRNA promoter (tRNA^Gly^) from *C. tropicalis* ATCC 20336, which functions as an RNA polymerase III promoter, demonstrating a more refined approach adapted to the cellular context of the species. The tRNA:gRNA fusion platform showed significant advantages, including broad applicability across different hosts due to the highly conserved nature of the tRNA processing system, and the ability to express multiple gRNAs from a single polycistronic gene, enabled by the small size of tRNAs (> 90 nucleotides). This advance represents an important transition from earlier approaches that relied on heterologous promoters and limited target multiplexing.

In addition, Li et al. (2022) introduced a dCas9‐based CRISPRi system for *C. tropicalis*, expanding the use of CRISPR beyond permanent genome editing. When combined with the tRNA:gRNA platform, CRISPRi enabled fine‐tuned regulation of gene transcription by targeting regions upstream or downstream of the start codon, thereby repressing target gene expression. The CRISPRi system, composed of a catalytically inactive Cas9 (dCas9) and a sgRNA, acts through steric hindrance of RNA polymerase binding or progression when positioned at the promoter or within the coding region of a gene (Knott and Doudna 2018). The effectiveness of this approach was validated through repression of *ERG9*, leading to increased β‐carotene production. This study illustrates an important conceptual shift, whereby CRISPR in *Candida* is used not only as a genome‐editing tool but also as an instrument for dynamic control of gene expression.

This consolidation of the CRISPR/Cas9 platform in *C. tropicalis* enabled additional applications, as demonstrated by Zhang et al. (2022, 2024), who employed the system for the heterologous production of α‐humulene and cembratriene‐ol (CBT‐ol). Initially, CtCas9 (Cas9) expression was integrated into the *CAT* locus, followed by deletion of the *URA3* gene to generate the uracil‐auxotrophic strain Cu‐208, thereby establishing a versatile and reusable genome‐editing platform. For gene integrations, linearized plasmids and Cas9 constructs containing sgRNAs were introduced into Cu‐208, following previously established methodologies (Vyas et al. 2015; Zhang et al. 2022). This approach enabled precise integration of heterologous synthase genes, such as *ZSS1* and *CBTS1*, as well as rational optimization of metabolic pathways through adjustment of gene dosage for key targets (*ERG20*, *BTS1*, and *E10*). These results highlight the maturity of the CRISPR system in *Candida*, which is now capable of supporting iterative metabolic engineering cycles with high genetic stability.

The versatility of these tools was further demonstrated in the study by Wei et al. (2024), in which both CRISPR/Cas9‐based gene integration and the CRISPRi system were applied in *C. tropicalis* DC03 to enhance squalene biosynthesis. The authors combined multiple strategies, including promoter modulation, replacement of endogenous genes with orthologs from *S. cerevisiae*, increased gene copy number, construction of fusion genes (*ERG20–ScERG9*), CRISPR/Cas9‐mediated gene deletions (*DPP1*, *BTS1*, and *ERG1*), and transcriptional repression of *ERG1* via CRISPRi. This combinatorial approach reflects an advanced stage of CRISPR engineering in *Candida*, in which the tool is synergistically integrated with classical metabolic engineering strategies. As a result, the *C. tropicalis* CS19E strain achieved 433.06 mg/L of squalene in a 30 L fermenter.

Finally, the expansion of CRISPR/Cas9 use to less explored species within the genus is exemplified by the work of Pham et al. (2023), who applied the system for the first time in *C. viswanathii* to enhance dodecanedioic acid (DDA) production. In this study, system standardization required the evaluation of six endogenous promoters, highlighting the ongoing need for species‐specific adaptation of CRISPR tools in *Candida*. The pTDH1pr was selected for sgRNA expression, while pPGK1 was used for SpCas9 expression. A multifunctional vector was constructed containing Cas9 expression cassettes for SpCas9, sgRNA, and the resistance gene *NrsR*. Two sgRNAs targeted the *Ade2* gene, whose disruption results in pink colonies, enabling phenotypic validation of editing. After standardization, four genes (*CYP52A19*, *CPRb*, *FAO2*, and *POS5*) were cointegrated, resulting in a stable strain that doubled DDA production, reaching 224 g/L. This work demonstrates that, although CRISPR/Cas9 in *Candida* has reached a high level of sophistication in model species, its application in non‐conventional species still requires initial optimization efforts, reinforcing the importance of systematic and comparative approaches for the continued advancement of the methodology.

## 4 Challenges for Applying CRISPR/Cas9 in Oleaginous Yeasts

**Figure 2:**
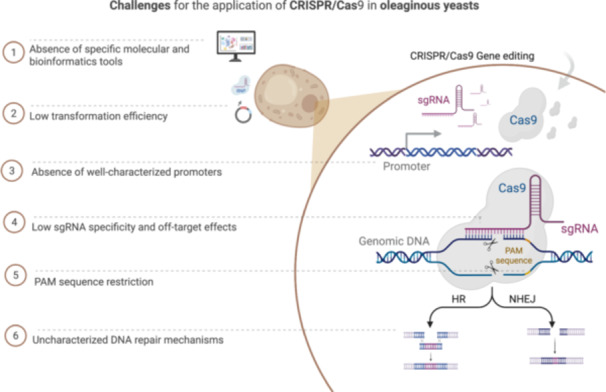
Challenges for the application of CRISPR/Cas9 in oleaginous yeasts.

Despite advances in the application of the CRISPR/Cas9 system in *S. cerevisiae*, the establishment of a standardized and efficient platform for non‐conventional yeasts faces obstacles that reflect the greater complexity and diversity of these species compared to the model yeast *S. cerevisiae* (Figure 2) (Raschmanová et al. 2018). Supporting Information Material S1 provides a comparative analysis of the developmental status of CRISPR–Cas tools in *S. cerevisiae*, the model oleaginous yeast *Y. lipolytica*, and non‐conventional oleaginous yeasts, highlighting pronounced differences in genome‐editing efficiency, predominant DNA repair mechanisms, and the availability of Cas nuclease variants. While *S. cerevisiae* exhibits high editing efficiency, a predominance of HR, and a broad repertoire of advanced strategies—such as multiplex editing, CRISPRi/a, and prime editing—oleaginous yeasts such as *Y. lipolytica* and non‐conventional species, including *Candida* spp., *R. toruloides*, and *C. oleaginosus*, rely primarily on NHEJ, require longer homology arms, and display variable or limited editing efficiency. Moreover, the scarcity of bioinformatic tools, the limited exploration of off‐target effects, and the incomplete consolidation of multiplex editing strategies in these yeasts further reinforce the technical challenges associated with standardizing CRISPR platforms (Hu et al. 2024; Wu et al. 2025).

One of the main challenges is the low transformation efficiency, which hinders the introduction of the necessary CRISPR/Cas9 components for gene editing. This issue can be attributed to the unique composition of each yeast's cell wall, which is often thicker or less permeable, as well as limitations in existing protocols, such as electroporation or chemical transformation methods. To overcome these challenges, researchers have focused on transformation techniques based on the removal of the yeast cell wall and the formation of spheroplasts, which exhibit high transformation efficiency and genetic material incorporation (Shaigani et al. 2023).

Efficient and stable expression of both Cas9 and sgRNA can be a drawback in non‐conventional yeasts, mainly due to the lack of well‐characterized and species‐specific promoters. Furthermore, many of these species have distinct codon preferences, which can reduce the translation efficiency of heterologous proteins, such as Cas9, requiring codon optimization to maximize functional expression (Nguyen et al. 2024). Proper expression of sgRNAs is also hindered by the lack of appropriate promoters, making it essential to identify strong regulatory elements compatible with the transcriptional regulation systems of the species of interest. These limitations reinforce the need of developing custom strategies, based on identifying native promoters and, when necessary, constructing synthetic promoters using promoter engineering, to create more efficient solutions that can circumvent some of the barriers associated with genetic expression (Nguyen et al. 2024).

Additionally, both identification and optimization of the PAM sequence necessary for Cas9 activity are limited, restricting the available editing sites. To address this issue, it is necessary to explore a broad range of Cas9 proteins, identifying characteristics that can reduce PAM recognition specificity, thus increasing the number of recognition sequences (Karvelis et al. 2017). Another critical issue is the difference in DNA repair mechanisms. Many non‐conventional yeasts rely more on mechanisms like NHEJ rather than HR, which reduces the accuracy of genomic editing and makes it harder to introduce specific mutations or integrate exogenous genes (Bai et al. 2023). Moreover, the lack of specific molecular and bioinformatic tools for non‐conventional yeasts, as well as the availability of genetic libraries and well‐annotated genomes, imposes additional challenges. Developing custom strategies is crucial to overcome the intrinsic limitations of each species.

Among these challenges, the need for the implementation of standardized transformation methods for unconventional yeasts, which are still poorly characterized, stands out. These methods should explore the use of lytic enzymes, such as lyticase, zymolyase, glucanases, and proteases, in order to reduce the physical barrier imposed by the cell wall. These strategies include protoplast‐mediated transformation approaches, as well as the engineering development of species‐specific promoters capable of maximizing the expression of Cas9 and sgRNA. Additionally, the exploration of Cas9 variants with less restrictive PAM requirements emerges as a promising alternative to expand the number of editable targets and increase the efficiency of genome editing in unconventional species.

Another challenge is the lack of endogenous free plasmids, as evidenced in *Rhodotorula* spp. Additionally, the absence of autonomous chromosomal replication sequences (ARS) complicates the stable maintenance of exogenous plasmids, making the use of self‐replicating vectors unfeasible for successive genomic edits or long‐term gene expression. This necessitates the use of integrative plasmids, which can lead to modified strains with editing scars. Finally, HR, essential for targeted gene replacements, exhibits low efficiency, with most studies relying on NHEJ (Table 1), reducing the precision of genomic edits. Circumventing these challenges is crucial to enhance CRISPR/Cas9‐mediated gene editing in the *Rhodotorula* genus and harness its potential as an industrial platform. These efforts are pivotal to expand the application of non‐conventional yeasts in bioprocesses.

## 5 Conclusion and Perspectives

The advancement of CRISPR‐Cas9 technology in oleaginous yeasts, along with other metabolic engineering strategies such as adaptive laboratory evolution and HR, has paved the way for optimizing lipid production and formulating customized fatty acids for various industrial purposes. Expanding the use of the CRISPR system to other oleaginous yeasts, beyond *Y. lipolytica* and *R. toruloides*, is pivotal to explore the biotechnological potential of other genera, such as *Lipomyces* spp and *Candida* spp. Even though different plasmids and expression strategies are currently available, an efficient approach for certain species may not be equally successful in others. Optimizing current CRISPR/Cas9 systems would contribute to the development of expression tools like hybrid promoters that are functional for controlling transcription processing in different species within the same genus. Furthermore, expanding the range of Cas endonucleases with diverse functions in genome editing and gene expression control—including, in addition to Cas9, systems such as Cas12a (Cpf1), Cas12b, Cas13, and Cas14, as well as catalytically inactive or modified variants (dCas)—can significantly enhance the core CRISPR toolkit, providing the potential to replace less targeted traditional techniques and enable more precise and versatile gene modulation. However, despite their increasing use in model organisms, the application of these alternative systems does not exist in non‐causal oleaginous yeasts, reflecting limitations related to efficient delivery, functional expression of the nucleases, and the lack of systematic standardization studies in these species. The construction of sgRNAs based on hybrid systems with tRNA under the control of conserved tRNA promoters could provide greater stability and transcription efficiency in yeasts belonging to the same order. The CRISPR‐Cas9 expression strategies addressed in this review offer a snapshot of the current landscape, contributing to the development of new strategies. In the coming years, CRISPR‐Cas9 tools using components shareable between different oleaginous yeasts will be designed, facilitating their rapid application across multiple species and opening new avenues for metabolic engineering.

## Author Contributions

Rodrigo Gonçalves Dias, Fernanda Pinheiro Freitas, Eduardo Luís Menezes de Almeida, and Wendel Batista da Silveira defined the scope of the review. Rodrigo Gonçalves Dias conducted the literature survey and drafted the manuscript and figures. Rodrigo Gonçalves Dias, Fernanda Pinheiro Freitas, Eduardo Luís Menezes de Almeida, Luciano Gomes Fieto, Agustin Zsögön, and Wendel Batista da Silveira contributed to the critical revision and editing of the final manuscript.

## Ethics Statement

The authors have nothing to report.

## Consent

The authors have nothing to report.

## Conflicts of Interest

The authors declare no conflicts of interest.

## Take Aways

- Few efficient CRISPR/Cas platforms are available for non‐conventional yeasts.
- Bioinformatic tools for non‐conventional oleaginous yeasts remain scarce.
- Efficient expression of Cas9 and sgRNA is still a major technical bottleneck.
- Limited knowledge of genetic DNA repair mechanisms hampers CRISPR standardization.

## Supporting information

Supporting Material 1: Comparative overview of CRISPR–Cas9 platforms and genome editing features in model and non‐conventional yeasts.

## Data Availability

The data supporting the findings of this study are available within the article and its supporting material. No additional data sets were generated or analyzed during the current study.
