# Supplementary material for: CRISPR/Cas9 Genome Engineering in Non‐Conventional Oleaginous Yeasts: Applications, Challenges, and Prospects
Source: Yeast. 2026 Mar 19;43(3):77–88. doi: 10.1002/yea.70015 (PMC13159405; doi:10.1002/yea.70015)
Supplement: Supplementary file 1 — Supporting Material 1: Comparative overview of CRISPR–Cas9 platforms and genome editing features in model and non‐conventional yeasts. [file YEA-43-77-s001.docx]

Supplementary Material 1: Comparative overview of CRISPR–Cas9 platforms and genome editing features in model and non-conventional yeasts.

| **Parameter** | ***Saccharomyces cerevisiae*** | ***Yarrowia lipolytica*** | ***Candida* spp*.*** | ***Rhodotorula toruloides*** | ***Cutaneotrichosporon oleaginosus*** |
| --- | --- | --- | --- | --- | --- |
| **Organism status** | Model yeast | Model oleaginous yeast | Non-conventional yeasts (CTG clade) | Non-conventional oleaginous yeast | Emerging non-conventional oleaginous yeast |
| **CRISPR–Cas9 efficiency** | Very high (>80–95%) | High but variable (30–90%) | Variable, species-dependent; clinically relevant species are more extensively explored | Moderate (10–70%) | Low to moderate (limited sample size) |
| **Predominant DNA repair mechanism** | HDR-dominant | NHEJ-dominant | NHEJ-dominant | NHEJ-dominant | NHEJ-dominant |
| **Efficient homology arm length** | Short (40–100 bp) | Long (≥100 bp–2 kb) | Long (≥500 bp–1 kb) | Long (≥1 kb) | Long (≥1 kb) |
| **Cas protein expression** | - SpCas9 – active Cas9 endonuclease (Streptococcus pyogenes)  - SaCas9 – active Cas9 endonuclease (Staphylococcus aureus)  -nSpCas9 (D10A) – Cas9 nickase (RuvC domain inactivated)  -nSpCas9 (H840A) – Cas9 nickase (HNH domain inactivated)  -dSpCas9 (D10A + H840A) – catalytically inactive Cas9 (dead Cas9)  -dCas9–CRISPRi – dCas9 for transcriptional repression  -dCas9–CRISPRa – dCas9 for transcriptional activation  -CBE (nCas9–cytidine deaminase–UGI) – cytosine base editor  -ABE (nCas9–adenine deaminase) – adenine base editor  -PE (nCas9–reverse transcriptase) – prime editing | -SpCas9 – active Cas9 endonuclease (Streptococcus pyogenes)  -nSpCas9 (D10A) – Cas9 nickase (RuvC domain inactivated)  -nSpCas9 (H840A) – Cas9 nickase (HNH domain inactivated)  -dSpCas9 (D10A + H840A) – catalytically inactive Cas9  -dSpCas9–Mxi1 – dCas9 fused to the transcriptional repressor Mxi1 (CRISPRi)  -dSpCas9–VPR – dCas9 fused to the tripartite activator VPR (CRISPRa)  Cas12a (Cpf1) – Cas12a/Cpf1 endonuclease (T-rich PAM, staggered cleavage) | -SpCas9 – active Cas9 endonuclease (Streptococcus pyogenes) | -SpCas9 – active Cas9 endonuclease (Streptococcus pyogenes) | -SpCas9 – active Cas9 endonuclease (Streptococcus pyogenes) |
| **Multiplex editing** | Well established and efficient | Variable efficiency | Rare | Limited | Scarce |
| **Off-target effects** | Rare due to well-established platforms | Poorly explored | Poorly studied | Poorly studied | Not studied |
| **Bioinformatic tools** | Extensive | Moderate | Limited | Scarce | Scarce |
| **Editing types** | Knockout; Knock-in; Single-nucleotide polymorphisms; Precise genomic integration | Knockout; Knock-in | Predominantly knockout | Knockout | Knockout |
| **Main applications** | Lipids, β-carotene, free fatty acids, lactate, 3-HP, BDO, and mogrol | Lipids, terpenoids | Lipid production and clinical/pathogenicity studies | Lipids, carotenoids, biofuels | Lipid production from by-products |
| **Reference** | (Wu et al., 2025) | (Hu et al., 2024) | Uthayakumar et al. (2021), Vyas et al. (2015), Li et al. (2022), Knott & Doudna (2018), Zhang et al. (2020), Zhang et al. (2022), Zhang et al. (2024), Wei et al. (2024), Pham et al. (2023) | Zhang et al. (2024), Otoupal et al. (2019), Jiao et al. (2019), Schultz et al. (2019), Jiao et al. (2021), Song et al. (2023), Schultz et al. (2022), Liu et al. (2024a), Cao et al. (2022), Liu et al. (2024b), Lyu et al. (2024), Reķēna et al. (2025), Yu et al. (2025) | Shaigani et al., 2023) |
